# Efficacy, safety, and tolerability of soticlestat (TAK-935) as adjunctive therapy in pediatric patients with dravet syndrome and Lennox–Gastaut syndrome: a meta-analysis of 3 randomized controlled trials

**DOI:** 10.3389/fphar.2025.1586098

**Published:** 2025-04-23

**Authors:** Zhang Lanlan, Jiang Nana, Wang Chengzhong

**Affiliations:** The Affiliated Maternity & Child Health Hospital of Yangzhou University, Yancheng, China

**Keywords:** soticlestat, meta-analysis, DRAVET syndrome, lennox-gastaut syndrome, randomized controlled trial

## Abstract

**Purpose:**

To evaluate the efficacy, safety, and tolerability of soticlestat as adjunctive therapy in pediatric patients with epileptic encephalopathies of Dravet syndrome (DS) and Lennox-Gastaut syndrome (LGS).

**Method:**

We performed a computerized literature search of MEDLINE, EMBASE, Cochrane Library, Web of Science, Google Scholar, and ClinicalTrials.gov to identify eligible randomized, placebo-controlled trials (RCTs) until December 2024. We calculated risk ratios (RRs) for efficacy of responder rate, and tolerability profiles in terms of serious adverse event (SAE) and dropout for adverse events as well as the most common side effects. Quality assessment of included RCTs was performed using the Cochrane Collaboration tool.

**Results:**

A total of 3 RCTs with 553 patients were included in the current study. Pooled RR for responder was 3.88 (95% CI 1.78–8.49, P = 0.001) among patients with DS, and for patients with LGS was 1.56 (95% CI 0.91–2.68, P = 0.11). Significantly more patients receiving soticlestat experienced discontinuation than placebo (RR 2.82 1.49–5.33, P = 0.001) because of adverse events. No significant difference in SAE was observed between the two treatment groups with RR 0.87 (95% CI 0.55–1.39, P = 0.57). Among the most common AE, only constipation occurred more often in the soticlestat group (RR 3.71, 95% CI 1.22–11.31, P = 0.02).

**Conclusion:**

Soticlestat showed significantly higher efficacy in reducing convulsive seizures in patients with DS. Nonetheless, for patients with LGS, the difference between soticlestat and placebo was not statistically significant. The incidence of SAE in patients receiving soticlestat was similar to those receiving placebo; however, substantially more patients allocated to soticlestat discontinued prematurely because of side effects.

## Introduction

Developmental and/or epileptic encephalopathies (DEEs) are a group of conditions in which cognitive, sensory, and motor functions delay or regression because of epileptic activity (Scheffer and Liao, 2020). The clinical and EEG characteristics of DEEs vary widely, depending on the age at onset, and may change over time, according to the successive age ranges. According to the International League Against Epilepsy (ILAE) Commission on Classification and Terminology, epileptic encephalopathy is defined as “embodies the notion that the epileptic activity itself may contribute to severe cognitive and behavioral impairments above and beyond what might be expected from the underlying pathology alone and that these might worsen over time” (Berg et al., 2010). DEEs are associated with developmental delay and intellectual disability, leading to significant morbidity and quick deterioration of quality of life (McTague et al., 2016). Additionally, patients with DEE have an increased risk of sudden unexplained death in epilepsy (SUDEP), resulting in up to 17% of patients with DS die by age 20, predominantly due to SUDEP (Scheffer et al., 2024). Therefore, early diagnosis is often critical; however, seizures caused by DEEs are usually difficult to control, as they are highly resistant to most currently available antiseizure medications (ASMs).

Dravet syndrome is one of the most common pharmaco-resistant epilepsies, although being treated with multiple ASMs, it is estimated that up to 90% of patients cannot achieve complete seizure control (Sullivan et al., 2024). At present, only three ASMs (cannabidiol, fenfluramine, and stiripentol) have been approved by the FDA for the treatment of seizures associated with Dravet syndrome. However, these three ASMs showed limited efficacy in completely control seizure onset (De Liso et al., 2016). As for LGS, although six ASMs (lamotrigine, topiramate, felbamate, rufinamide, clobazam, and clonazepam) are approved in the USA and Europe for the treatment of LGS, similar to DS, more than 90% of seizures with LGS are poorly controlled and patients still experience disabling seizures (Douglass, 2011).

Soticlestat (TAK-935/OV935) is the first potent, selective, and CNS-penetrant inhibitor of cholesterol 24-hydroxylase (CH24H) being investigated as an adjunctive treatment for seizures associated with DEEs, including DS and LGS (Nishi et al., 2022). CH24H is primarily expressed in cortical and hippocampal neurons, where it facilitates the neuronal synthesis of 24S-hydroxycholesterol (24HC) (Nishi et al., 2020). By inhibiting CH24H and reducing 24HC levels, soticlestat is proposed to decrease seizure frequency and severity, offering potential therapeutic benefits for epilepsy and other disorders linked to overactive glutamatergic signaling (Wang et al., 2021; Wang et al., 2022). Recently, several RCT results of soticlestat for the treatment of DS and/or LGS have been published, this study intended to summarize currently available evidence of efficacy, safety, and tolerability for pediatric patients with DS or LGS.

## Materials and methods

This systematic review and meta-analysis was carried out in compliance with the Preferred Reporting Items for Systematic Reviews and Meta-Analyses (PRISMA) Statement and the Cochrane Handbook (Liberati et al., 2009; Higgins and Green, 2008). The primary outcome was the responder rate (50% reduction of seizure onset frequency from baseline) of convulsive seizures in LGS and drop seizures in DS during the treatment period. Secondary outcomes included dropout rate for any reason and adverse events, along with tolerability profiles in terms of dropout rate and the incidence of SAE. In addition, the most common adverse events reported by at least 2 RCTs were analyzed.

### Search strategy

A systematic literature search of MEDLINE (Ovid and PubMed), Embase, Cochrane Library, Web of Science, and Google Scholar from inception until 30 June 2024, with no language restriction applied. An additional search was performed to retrieve trials reporting results from the US National Institutes of Health database of clinical trials19 and the World Health Organization International Controlled Trials Registry website. We used the following combination of search terms: (“soticlestat” OR “TAK-935” OR “OV935”) AND (“seizure” OR “epilepsy” OR “epileptic encephalopathies” OR “DS” OR “LGS”). We also inspected references from the most recent reviews.

### Inclusion and exclusion criteria

We included studies that met all of the following criteria: patients diagnosed with DS or LGS; the use of soticlestat as adjunctive treatment for controlling seizure onset. We excluded studies if they satisfy any of the following criteria: trials conducted in the context of electroconvulsive therapy or surgery, retrospective or observational studies or with participants less than 20; not for treatment of DS or LGS but other DEEs such as seizures in tuberous sclerosis complex; without sufficient data to evaluate the efficacy; reviews, letters, comments, editorials, or conference abstracts.

### Data extraction and quality assessment

A predefined protocol was used to extract the following clinical information: authors, trial conducted sites, publication year, sample size of soticlestat and comparator group and corresponding dose, male/female ratio, participant age, treatment and follow-up period, outcomes in terms of number of responder and ≥75% reduction of seizure frequency from baseline, along with safety and tolerability such as dropout for any reason and side effect and incidence of SAEs. The Cochrane Collaboration’s Risk of Bias Tool was used to assess the risk of bias for each RCT (Higgins et al., 2011). Two reviewers independently conducted the data extraction and quality assessment, and disagreements were resolved through discussion.

### Data synthesis and analysis

Regarding treatment efficacy, we calculated the risk ratio and corresponding 95% CI for ≥50% (responder rate), ≥75%, and 100% reduction in seizure frequency (convulsive seizure for DS and drop seizure for LGS) from baseline. Concerning tolerability, we pooled the RRs for serious adverse events and dropout, as well as the most common adverse events that were reported by at least 2 RCTs. Besides, Changes in the investigator- and caregiver-reported Clinical Global Impression of Change (CGI-C) and Care GI-C scores on the impression of the efficacy and tolerability of treatment were calculated. Heterogeneity across included RCTs was assessed using ***Q*** statistics and inconsistency index ***I*** ^2^: 0%–40%, slight; 30%–60%, moderate; 50%–90%, substantial; and 75%–100%, considerable (Higgins and Green, 2008). All analyses were conducted using STATA, version 15.2 (StataCorp, Texas, United States), with P < 0.05 indicated statistically significant.

## Result

### Literature search

**FIGURE 1:**
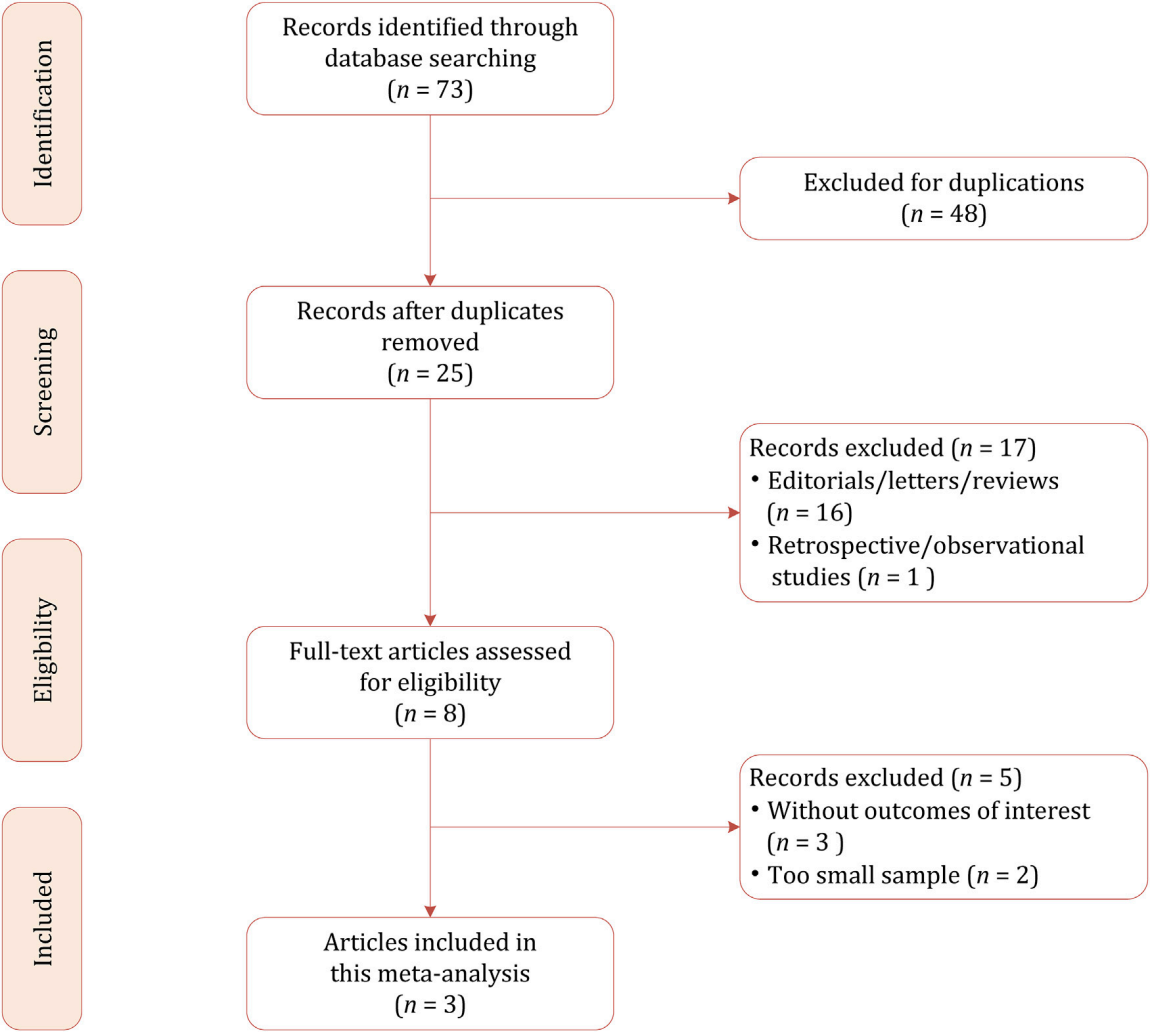
Study selection process for this meta-analysis.

Based on our literature search strategy, a total of 73 references were identified, out of which 48 were excluded for duplication. After examining the titles and abstracts, 22 results were excluded because they were not relevant to this meta-analysis due to the focus on pharmacokinetics. We performed full-text reviewing among the remaining articles and finally, a total of 3 RCTs involving 553 patients were included in this meta-analysis, of whom 276 were allocated to soticlestat and 277 were allocated to placebo (Takeda, 2025; Hahn et al., 2022; Takeda, 2024). The flow chart of the literature selection process is demonstrated in Figure 1.

### Study characteristics

The primary demographic and study characteristics are shown in Table 1. Of 3 RCTs, the earliest was registered on 27 August 2018 (NCT03650452), whereas the two others were registered on 18 June 2021 (NCT04938427) and 28 October 2021 (NCT04940624). 1 RCT reported the onset frequency of convulsive seizures during the baseline period for the DS group and drop seizures for LGS, however, two RCTs retrieved from ClinicalTrials.gov did not report the details on baseline. 1 study reported that for patients weighing ≥60 kg, soticlestat was administered up to 600 mg/day, with weight-based dosing used for those weighing <60 kg; 2 other RCTs reported that for patients <45 kg, soticlestat was administered up to 300 mg/day, whereas for patients ≥45 kg, the dose ranged from 40 to 200 mg/day. For all 3 RCTs, the overall treatment period included 4 4-week baseline, 4–8 weeks dose-optimization period, and 12 weeks maintenance period.

**TABLE 1:** Demographic characteristics of included trials.

Study	Location	DEE	Publication Year	Age (Mean ± SD)	Gender (M/F)	Number (STL/PBO)	Dosage (per day)	Primary endpoint	Treatment period
Hahn et al. (NCT03650452)	35 sites	DS and LGS	2022	8.7 ± 3.92/8.8 ± 4.50; 10.0 ± 4.19/9.8 ± 3.58	31/20 58/30	26/25 for DS 43/45 for LGS	≥60 kg, up to 300 mg BID; <60 kg, based on body weight	Reduction in convulsive seizures for DS and drop seizures for LGS	8-week titration +12-week maintenance
NCT04938427 (LGS)	84 sites	LGS	2024	13.4 (9.35) 12.5 (6.68)	163/107	134/136	<45 kg, 40–200 mg BID ≥45 kg, 100–300 mg BID	Reduction in drop seizures	4-week titration +12-week maintenance
NCT04940624 (DS)	multiple sites	DS	2025	10.1(5.04) 10.5(5.06)	72/72	73/71	<45 kg, 40–200 mg BID ≥45 kg, 100–300 mg BID	Reduction in convulsive seizures	4-week titration +12-week maintenance

Abbreviations: DDE, developmental and epileptic encephalopathies; DS, dravet syndrome; LGS, Lennox-Gastaut Syndrome; PBO, placebo; NA, not available; SD, standard deviation; STL, soticlestat.

### Quality assessment for included RCTs

1 of 3 RCT were deemed at low risk of bias for all 7 domains (Hahn et al., 2022), as for the remaining 2 RCTs retrieved from CinicalTrials.gov, the allocation concealment domain was considered as “unclear” because the detailed methods could not be obtained (Takeda, 2025; Takeda, 2024). Overall, the quality assessment of the included 3 RCTs was moderate.

### Efficacy for seizure onset frequency

**FIGURE 2:**
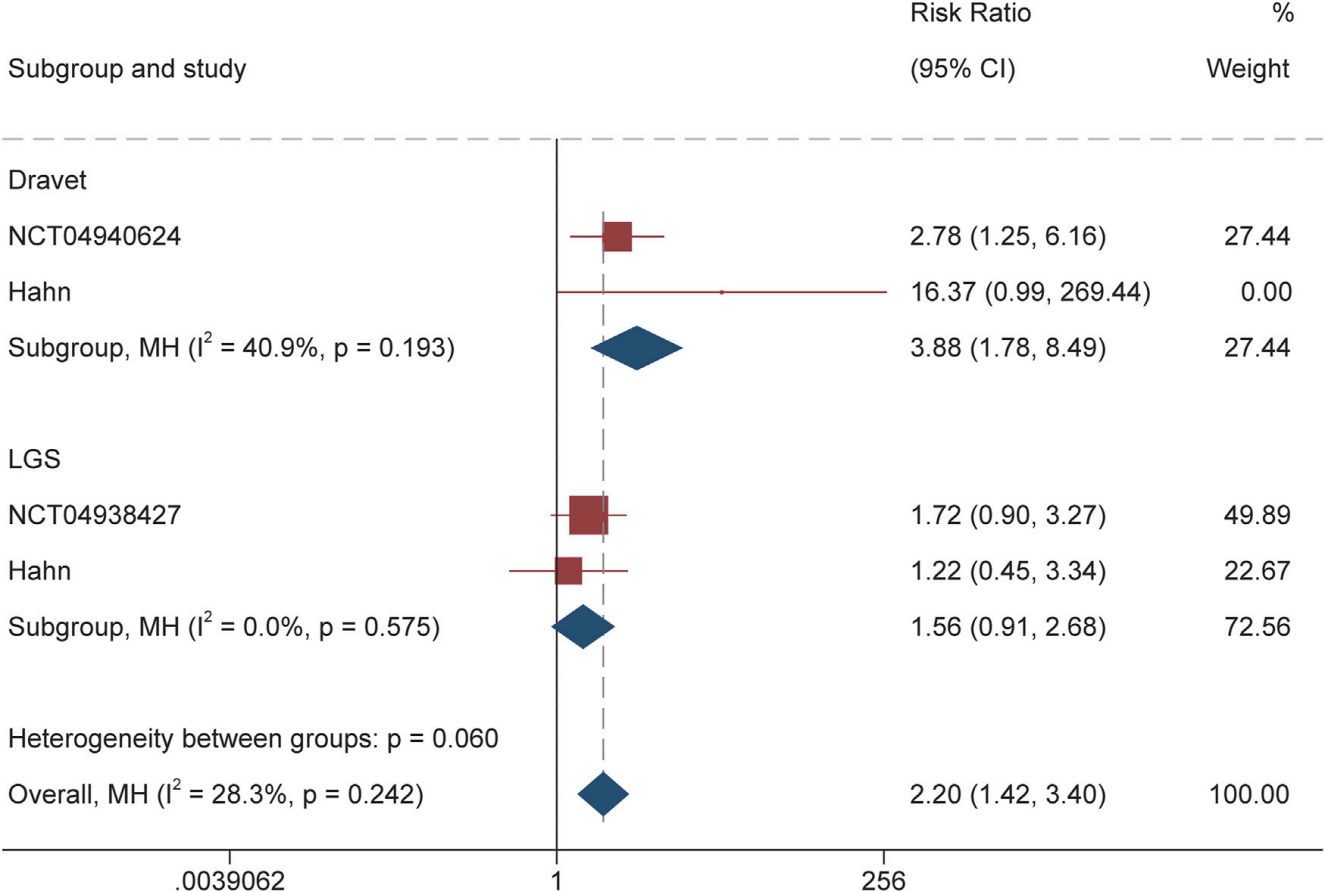
Forest plot of responder rate.

**FIGURE 3:**
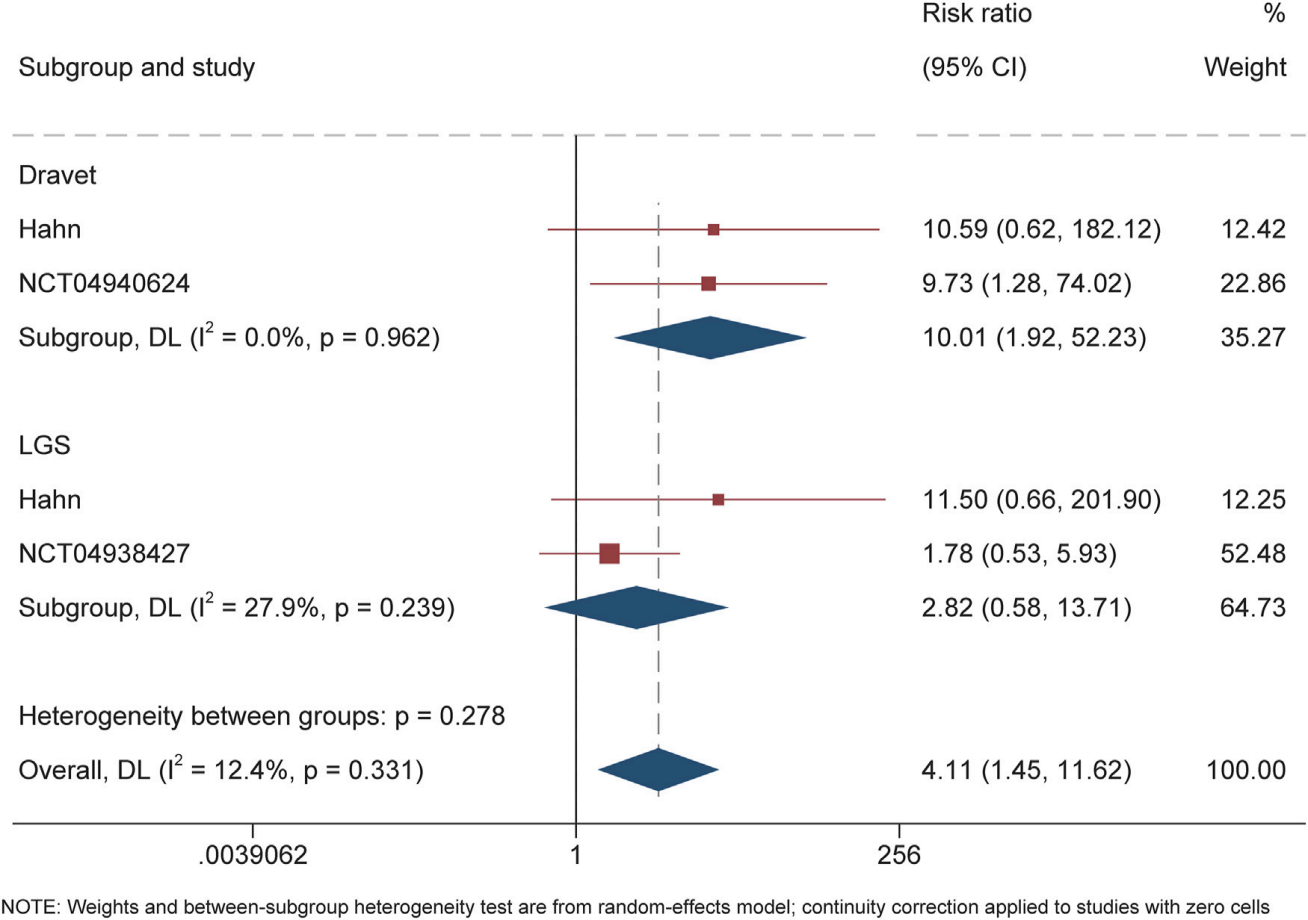
Forest plot of ≥75% seizure frequency reduction from baseline.

For DS, the calculated RR for the responder was 3.88 (95% CI 1.78–8.49, P = 0.001) throughout the overall treatment period (Figure 2). During the maintenance period, RR of 3.77 (95% CI 1.82–7.84, P < 0.001) also suggested the significantly higher proportion of patients receiving soticlestat than placebo achieving response. Similarly, RR of 10.01 (95% CI 1.92–52.23; P = 0.01) demonstrated that significantly higher proportion of patients receiving soticlestat than placebo had ≥75% reduction of seizure frequency (Figure 3). One RCT reported complete freedom (convulsive seizure) in 1 patient receiving soticlestat, whereas no patients in the placebo group achieved complete control of seizure. With regard to LGS, RR of 1.56 (95% CI 0.91–2.68, P = 0.11) suggested that no significant difference between the two treatment groups throughout the full treatment period. During the 12-week maintenance period, however, the pooled RR of 1.70 (95% CI 1.04–2.79, P = 0.04) showed that soticlestat had higher efficacy than placebo. As for the outcome of ≥75% reduction frequency in drop seizures, pooled RR of 2.82 (95% CI 0.58–13.71; P = 0.20) demonstrated no difference between the two treatment groups. No RCT reported seizure freedom for the treatment of patients with LGS.

### Tolerability and side effects

**FIGURE 4:**
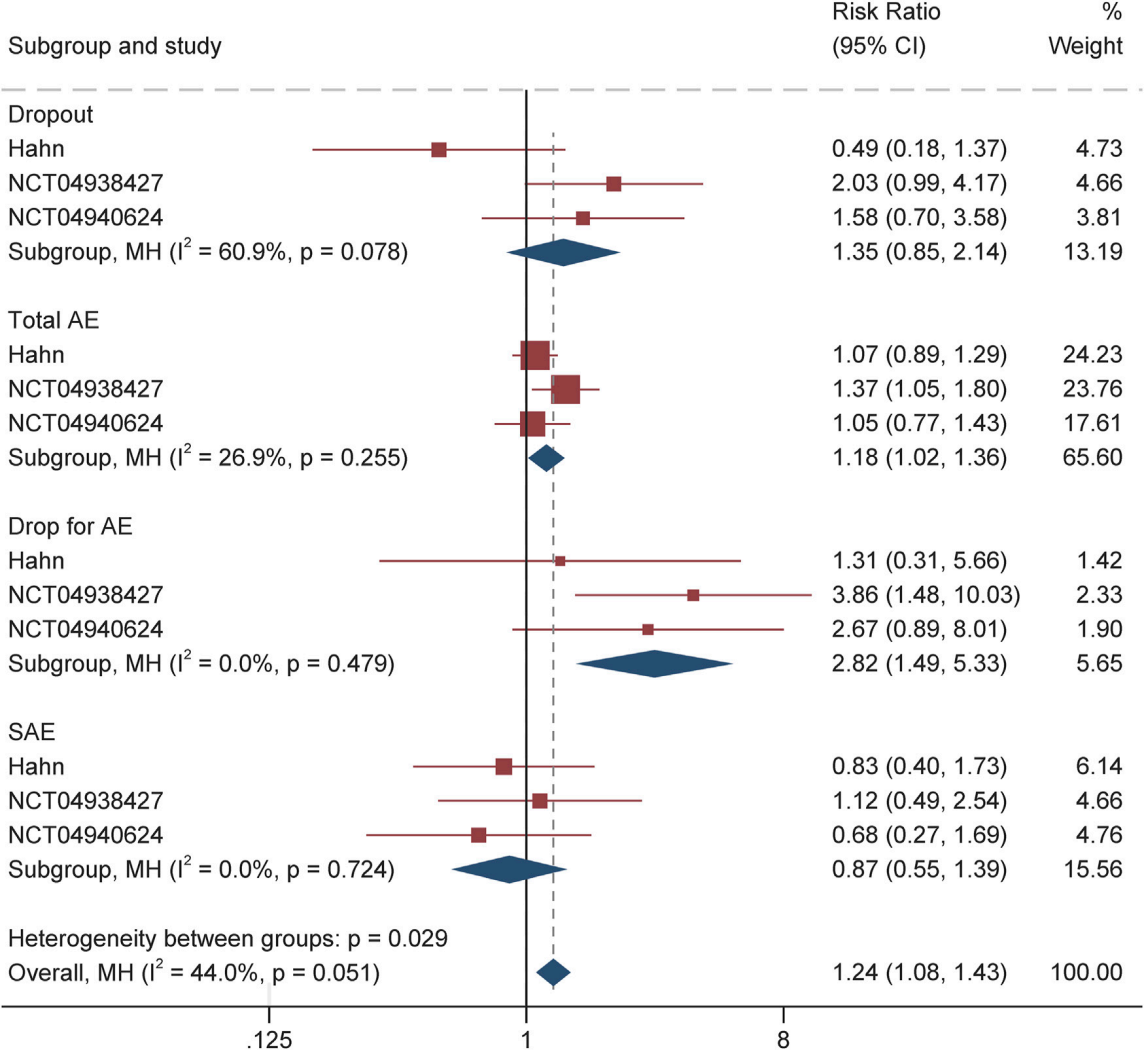
Forest plot of safety and tolerability profiles.

The safety and tolerability profile of soticlestat during the treatment period is summarized in Table 2 and Figure 4. Overall, the incidence of treatment-emergent adverse events among the soticlestat group was higher than placebo, with RR 1.18 (95% CI 1.02–1.36, P = 0.03). The proportion of patients who prematurely discontinued was higher among patients receiving soticlestat compared to placebo (RR 1.35, 95% CI 0.85–2.19); however, no significant difference was found between the two groups (P = 0.20). Nevertheless, substantially more patients allocated to soticlestat experienced early dropout due to adverse events (RR 2.82, 95% CI 1.49–5.33, P = 0.001). There was no significant difference in SAE between the two treatment groups (RR 0.87, 95% CI 0.55–1.39, P = 0.57); however, 1 RCT reported 1 mortality in the soticlestat group because of sudden unexplained death in epilepsy.

**TABLE 2:** Adverse and safety profiles.

Adverse event	Soticlestat (*N* = 278)	Placebo (*N* = 277)	Risk ratio (95% CI)*	*P*
Any AE	164	140	1.18 (1.02–1.36)	0.03
Serious adverse events	29	33	0.87 (0.55–1.39)	0.57
Dropout for Any Reason	38	28	1.35 (0.85–2.19)	0.20
Dropout for AEs	34	12	2.82 (1.49–5.33)	0.001
Fatigue	14	12	1.18 (0.54–2.56)	0.68
Pyrexia	29	28	1.03 (0.63–1.69)	0.91
Nasopharyngitis	25	26	0.96 (0.57–1.61)	0.86
Change in seizure	33	25	1.24 (0.59–2.61)	0.57
Upper Respiratory Tract Infection	30	27	1.09 (0.67–1.79)	0.72
Decreased Appetite	19	12	1.53 (0.75–3.11)	0.24
Somnolence	35	21	1.65 (0.98–2.76)	0.06
Constipation	17	3	3.71 (1.22–11.31)	0.02

Abbreviations: AE, adverse event; CI, confidence interval.

*Calculated with Mantel–Haenszel fixed-effects model.

As demonstrated in Table 2 and Supplementary Figure S1, the most common adverse events reported by at least two RCTs include fatigue, pyrexia, nasopharyngitis, change in seizure, decreased appetite, somnolence, upper respiratory tract infection, and constipation. Most of these adverse events had similar incidences among patients receiving soticlestat and placebo. Nonetheless, the incidence of constipation was more often among the soticlestat group (RR 3.71, 95% CI 1.22–11.31, P = 0.02). According to the CGI-C and Care CI-C score, investigator-reported improvement included “marked improvement and no side effects” and “marked improvement and minimal side effects”; caregiver-reported improvement included “slightly improved”, “much improved”, and “very much improved”. The pooled CGI-C (OR 2.17, 95% CI 1.36–3.47; P = 0.001) and Care CI-C (OR 1.72, 95% CI 1.03–2.87; P = 0.04) for LGS suggested significant improvement as compared with placebo. Likewise, these two outcomes of global functioning for DS also showed substantial improvement, with pooled CGI-C of OR 3.14 (95% CI 1.64–6.00; P = 0.001) and Care CI-C of OR 2.84 (95% CI 1.56–5.17; P = 0.001), which are demonstrated in Supplementary Figure S2.

## Discussion

In the present study, we performed a meta-analysis of the efficacy and tolerability of soticlestat administered orally or via gastrostomy tube/percutaneous endoscopic gastrostomy tube twice daily for the treatment of DS or LGS. Based on 3 high-quality RCTs, the pooled summary estimates showed the promising efficacy of soticlestat for DEEs of DS. During the 20 weeks of full treatment period, a significantly greater proportion of patients assigned to soticlestat than those receiving placebo achieved ≥50% reduction of convulsive seizure from baseline. However, for LGS, no significant difference was observed between the soticlestat and placebo groups in terms of responder rate. Moreover, 2 RCTs reported that no significant difference between the two treatment groups was observed in median change from baseline. Regarding the ≥75% reduction in seizure frequency, soticlestat showed superior efficacy compared to placebo in DS patients; however, in LGS, no significant difference was observed between the soticlestat and placebo groups for this endpoint. For the endpoint of seizure freedom, only one RCT reported a patient with DS achieved complete control with soticlestat. Although soticlestat treatment reduced plasma 24HC levels in both DS and LGS patients, a significant reduction in seizure frequency was observed only in the DS group. Further studies are warranted to evaluate the efficacy of soticlestat in LGS, which may help clarify the disconnect between 24HC level reduction and seizure outcomes in these two treatment groups.

For tolerability and safety, it seems that soticlestat could be well-tolerated and showed mild to moderate side effects, and no significant difference in the occurrence of SAE between the two treatment groups (29 of 278 vs 33 of 277). However, it should be noted that one RCT reported a case of SUDEP in a participant receiving soticlestat. The proportion of patients assigned to soticlestat discontinued premature was higher than those receiving placebo; however, the difference was not statistically significant. Nevertheless, sustantially more patients experienced premature discontinuation due to AE than those who received placebo. Among the most common side effects reported by more than 2 RCTs, only constipation had a higher incidence in patients receiving soticlestat, and most of the side effects were mild or moderate and transient. However, the pooled RR of 1.65 (95% CI 0.98–2.76, P = 0.06) indicated that somnolence had a higher incidence in soticlestat.

In this study, heterogeneity between trials was not formally assessed due to the limited number of included RCTs. Nonetheless, several factors likely contributed to variability across studies, including differences in inclusion criteria, definitions of convulsive and drop seizures, and baseline seizure frequency. Additional sources of heterogeneity may stem from variations in participants’ age and follow-up duration. Assessing drop seizures in LGS is particularly challenging, as their clinical presentation and significance differ depending on characteristics such as posture and the extent of body involvement. These nuances underscore the importance of individualized treatment approaches tailored to each patient’s specific symptoms and response patterns. Furthermore, drug–drug interactions may substantially influence the efficacy and tolerability of ASMs. While preclinical studies have assessed the pharmacokinetics of soticlestat in healthy adults, its interactions with concomitant ASMs were not addressed in the RCTs (Wang et al., 2021; Wang et al., 2022). Additionally, variations in treatment duration and dosage could impact clinical outcomes, highlighting the need for future studies to explore these factors to evaluate the therapeutic profile of this new ASM.

Beyond DS and LGS, soticlestat has been explored as a potential treatment for other DEEs, including chromosome 15q duplication syndrome, cyclin-dependent kinase-like 5 (CDKL5) deficiency disorder (CDD), and tuberous sclerosis complex. Halford et al. reported that in a randomized controlled trial, soticlestat was well tolerated at doses up to 300 mg BID and led to a reduction in median seizure frequency in patients with LGS, DS, or tuberous sclerosis complex (Halford et al., 2021). In a phase 1b/2a trial, adjunctive therapy of soticlestat demonstrated a reduction in median seizure frequency over the study duration. However, among participants receiving concomitant perampanel treatment, three experienced increased seizure frequency, with two showing seizure exacerbation. While no known pharmacokinetic interaction exists between soticlestat and perampanel, the observed effects may stem from a pharmacodynamic interaction or the inherent variability of seizures in DEEs. Further studies with larger cohorts are necessary to explore this potential interaction. In an open-label study, Demarest et al. found that adjunctive soticlestat treatment reduced motor seizure frequency in patients with CDD and decreased overall seizure frequency across both patient groups studied (Demarest et al., 2023). Conversely, in patients with Dup15q syndrome, soticlestat treatment was associated with increased motor seizure frequency. These findings highlight the need for further research to determine the variability of treatment effects across different DEEs.

In a recent network meta-analysis, Lattanzi et al. evaluated eight RCTs involving stiripentol, cannabidiol, fenfluramine, and soticlestat for the treatment of convulsive seizures in DS (Lattanzi et al., 2023). The analysis suggested that fenfluramine may offer superior efficacy over other ASMs in reducing convulsive seizure frequency. However, conclusions regarding soticlestat were limited due to the inclusion of only one RCT. Beyond efficacy, the selection of ASMs must also consider potential drug-drug interactions, which can significantly influence both therapeutic effectiveness and tolerability. For the treatment of DS, several novel therapeutic strategies for DS are currently under investigation. Ongoing RCTs are assessing serotonin modulators such as clemizole, lorcaserin, and LP352. In parallel, advancements in precision medicine, including antisense oligonucleotides and gene therapy, offer promising avenues by targeting the genetic underpinnings of the disorder (Matricardi et al., 2023). While existing treatments largely focus on seizure reduction, comprehensive care must also address non-seizure-related comorbidities, which are critical for improving overall quality of life.

LGS is known to be a complex condition with a heterogeneous etiology and clinical features, characterized by evolving clinical features and significant resistance to pharmacological treatment (Cross et al., 2017). Notably, among patients with DEEs, LGS has the highest proportion of treatment-resistant cases, with one study reporting a 90% resistance rate (Resnick and Sheth, 2017). The condition profoundly affects the QoL of both patients and caregivers, with drop seizure control and seizure-free days being the most influential factors. Therefore, treatment strategies should emphasize the reduction of the most disabling seizure types rather than aiming for reducing seizure onset frequency (Auvin et al., 2025). Our analysis demonstrated that even though soticlestat did not demonstrate a statistically significant advantage over placebo in reducing seizures among LGS patients, assessments of global functioning including the CGI-C and Care CI-C revealed significant improvements. These findings suggest that soticlestat may still offer clinical benefits for individuals with LGS beyond seizure control. For LGS, drop seizures in LGS can be challenging to define due to their varied presentation, which depends on body involvement (e.g., whole-body, or head-only) and patient positioning (Pujar and Cross, 2024). These assessments, which consider the patient’s history, psychosocial context, symptoms, behavior, and functional impact, capture broader treatment effects beyond seizure control. The management of LGS demands a personalized, multifaceted approach due to its clinical complexity. The syndrome encompasses a wide range of seizure types, cognitive impairments, and etiologies, which necessitate highly individualized treatment plans. Effective care typically involves a combination of pharmacological therapy, dietary interventions, and, in some cases, surgical procedures (Auvin et al., 2025). Given the evolving nature of the disease, ongoing assessment and tailored modifications to treatment are essential for optimizing outcomes and addressing the broad spectrum of comorbidities associated with LGS.

Our study is not without limitations. First, only 3 RCTs were included in the present meta-analysis, which lowers the applicability of our study. Nevertheless, we have included all currently available evidence of soticlestat for the treatment of DS or LGS to date. Second, moderate heterogeneity was observed in our study; however, due to too few RCTs, we did not perform sensitivity analysis to investigate the heterogeneity. Third, our study only pooled data on convulsive seizures in DS and drop seizures in LGS, other seizure types were not analyzed.

## Conclusion

Soticlestat showed significant efficacy in reducing convulsive seizures in patients with DS. Nonetheless, for patients with LGS, the difference between soticlestat and placebo not statistically significant. The incidence of SAE in patients receiving soticlestat was similar to those receiving placebo; however, substantially more patients allocated to soticlestat discontinued prematurely because of side effects.
