# Supplementary figures and images for: Efficacy, safety, and tolerability of soticlestat (TAK-935) as adjunctive therapy in pediatric patients with dravet syndrome and Lennox–Gastaut syndrome: a meta-analysis of 3 randomized controlled trials
Source: Front Pharmacol. 2025 Apr 23;16:1586098. doi: 10.3389/fphar.2025.1586098 (PMC12055774; doi:10.3389/fphar.2025.1586098)

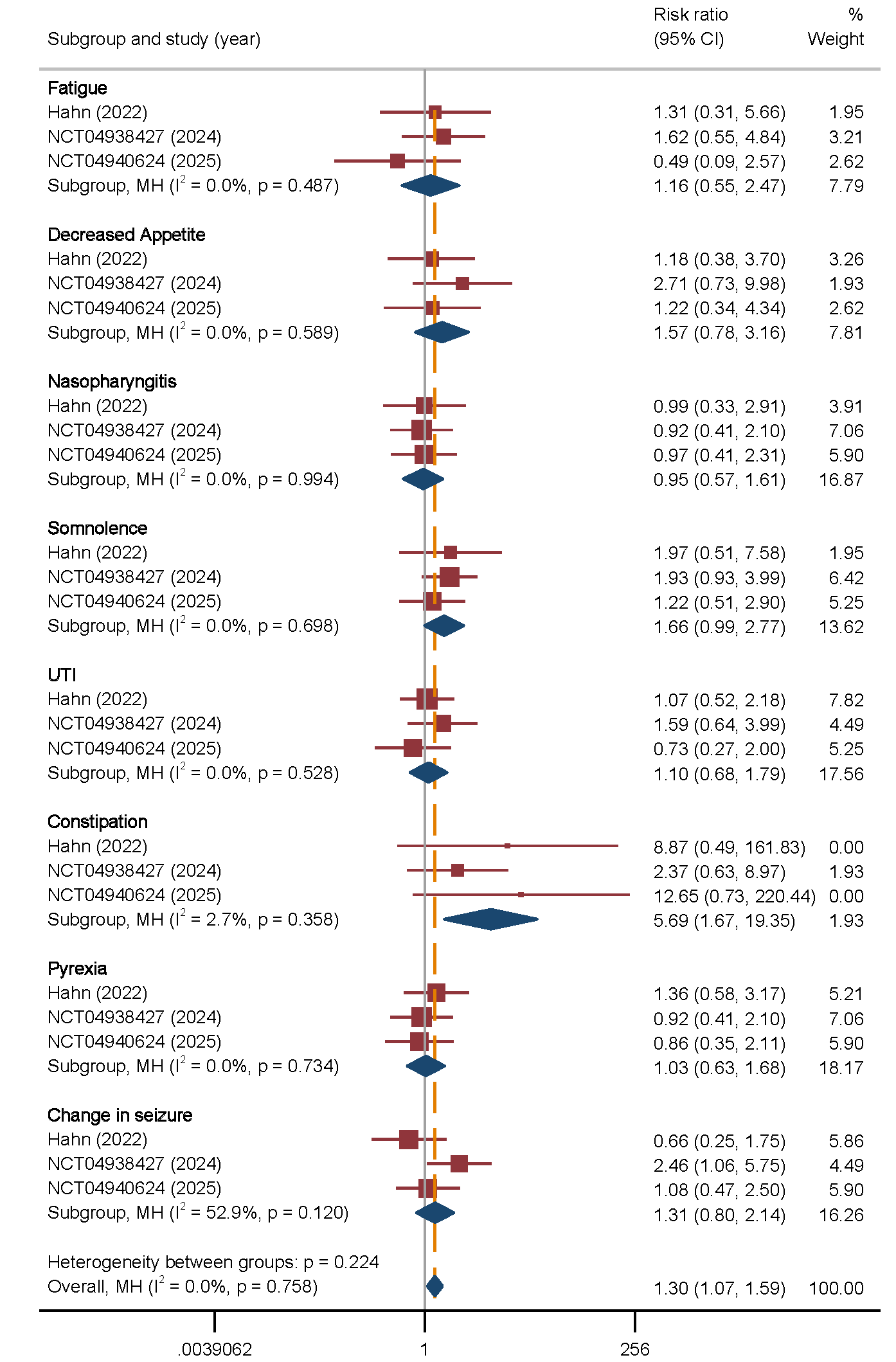

Supplement: Supplementary file 1 [file DataSheet1.zip › Supplementary Figure S1.tiff]

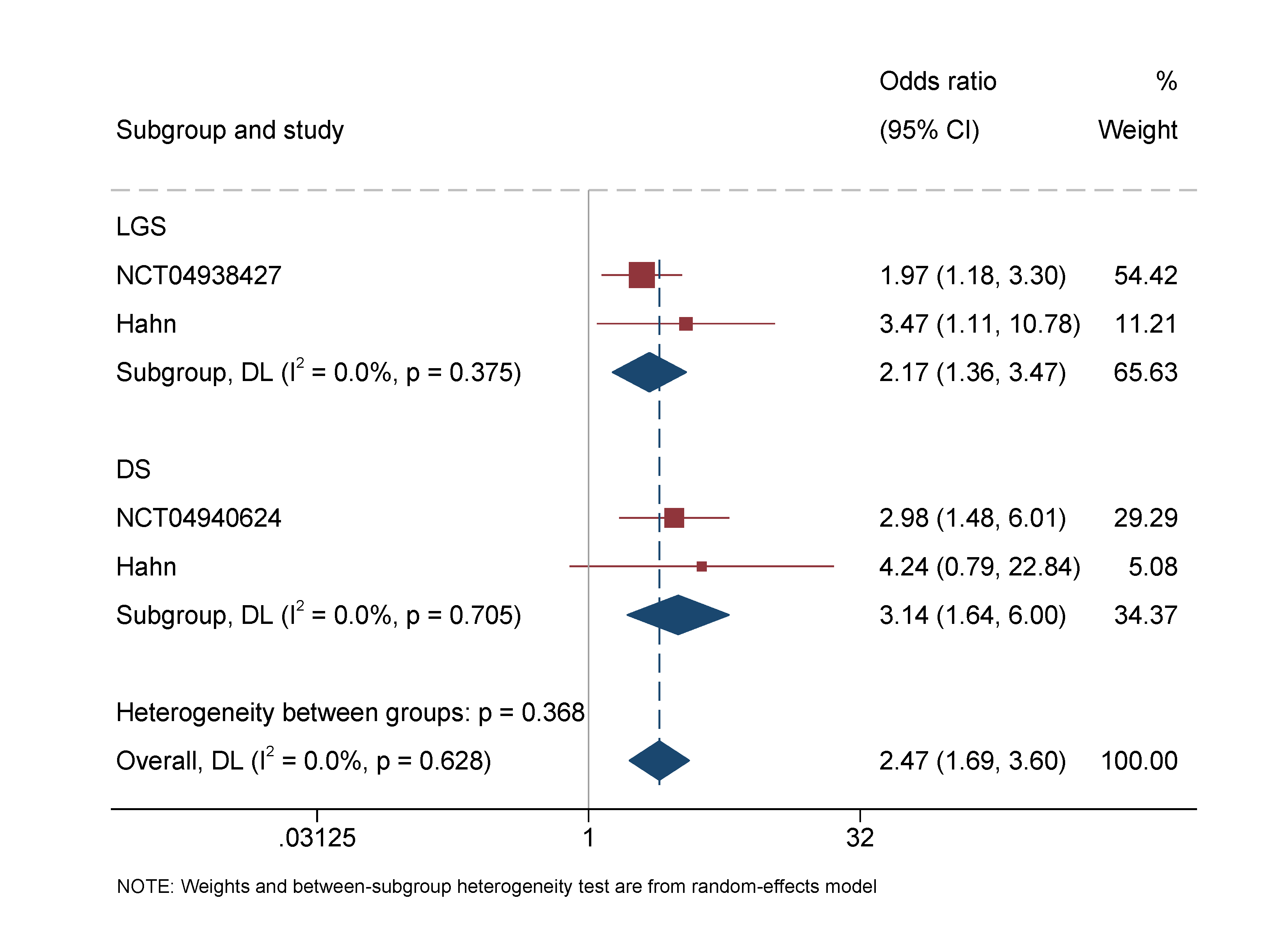

Supplement: Supplementary file 1 [file DataSheet1.zip › Supplementary Figure S2-A.tif]

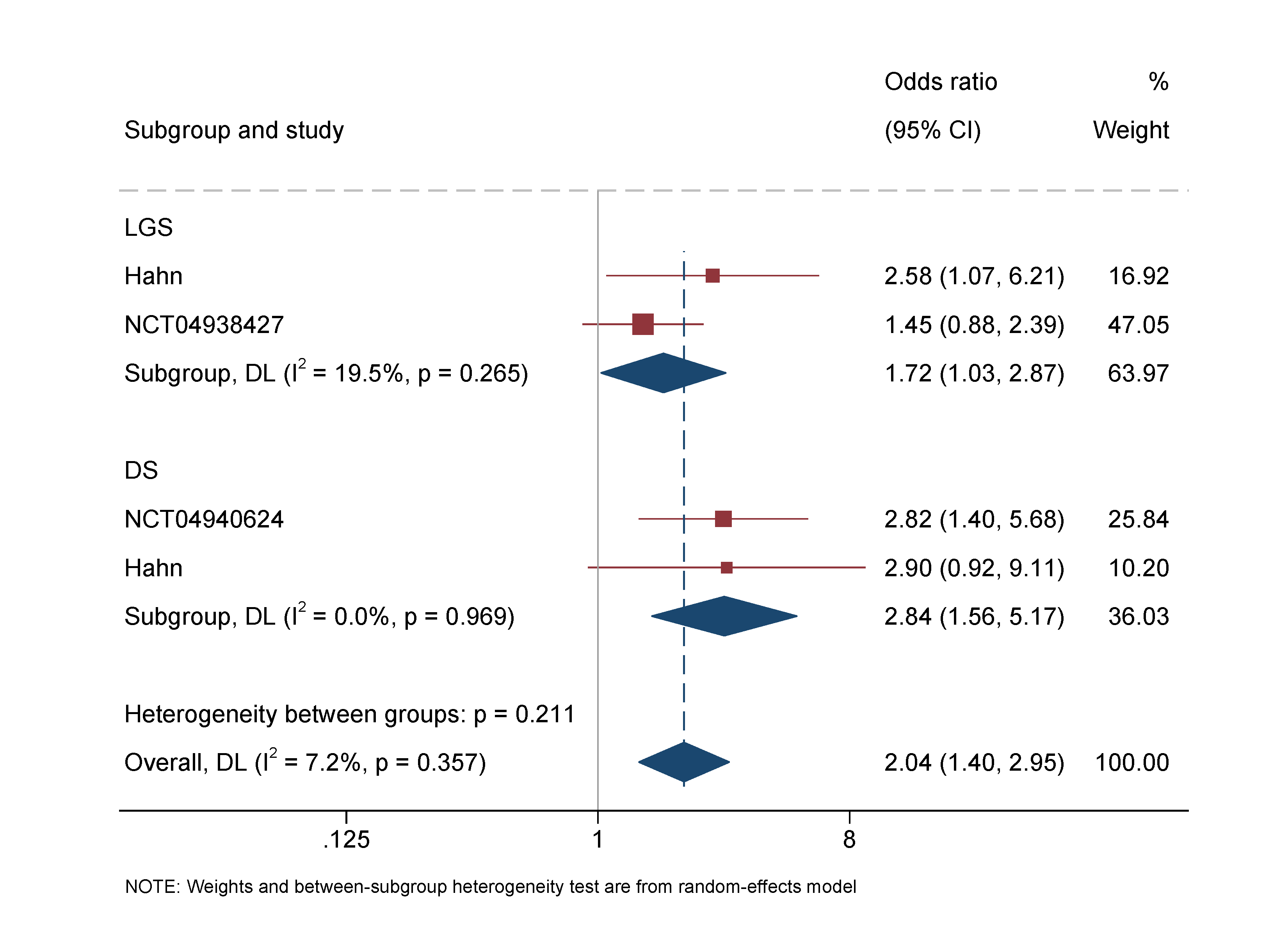

Supplement: Supplementary file 1 [file DataSheet1.zip › Supplementary Figure S2-B.tiff]
